# Surgery or Medicine?*Read before International Hahnemannian Association.

**Published:** 1885-01

**Authors:** Edward Rushmore

**Affiliations:** Plainfield, N. J.


### CLINICAL BUREAU.

### SURGERY OR MEDICINE?*

EDWARD RUSHMORE, M. D., PLAINFIELD, N. J.

CASE I.—Late last summer I was summoned to the bedside of a man of about fifty years, who could pass no urine, except a very few drops by the hardest straining. He was in the last stage of phthisis and near the close of life. No information could be elicited as to the probable cause of this retention. There was a sense of fullness and actual fullness in the hypogastrium, with frequent effectual urging. This condition had lasted from the previous day, an old school physician having vainly attempted to relieve him.

The use of the catheter suggested itself as the speediest means of relief, and it was attempted, but I could not introduce the instrument. Failing in this, I did what I ought to have done at first, looked for and administered the most homœopathic remedy. Cantharides was selected, and a few globules of Dr. Skinner's *Fluxion*, centesimal potency 20m, were dissolved in half a tumbler of water, and the patient directed to take a teaspoonful of the solution every twenty minutes until relieved, but to send for me if not relieved in two hours. I got no message from him, but on visiting him the next day I learned that he had passed his urine freely before the two hours had elapsed. He was never again troubled in that way.

CASE II.—*A Chronic Ulcer Cured.*—While on a short journey from home in the latter part of September, 1881, I met a gentleman from Brooklyn, who, on learning that I was a physician, asked me to look at an ulcer of more than two years' duration on his left leg. It was situated a little above the outer malleolus; was about as large as a quarter-dollar, with the areola much swelled, very hard, and of a dull, purplish red color. It was the seat of occasional stinging pains, and of a scanty, watery discharge. He had dressed it for a long time with what he called green ointment. I told him I thought it could be cured, but meeting him only transiently at the house of a mutual friend, had neither the time nor facilities for a full examination of the case and its related remedies. I gave him a high potency of

---

* Read before International Hahnemannian Association.

*Silicea*, requested him to remove all ointments from the sore, and if not better in a week or two, to come to see me if he should want me to do anything for him.

On November 2d, five weeks later, he visited me for the first time. The ulcer had not changed in appearance, but the stinging had given way to drawing sensations. Before the appearance of the ulcer he had much rheumatism, but none since the ulcer came. He feels generally better in winter and worse before a storm. The feet are hot at night; he keeps them out of bed and feels better with cold feet.

He received one dose of *Sulph.*$^{cm}$ (Fincke).

November 14th.—Has scarcely any pain in ulcer; the edges look contracting; less discharge; less heat in feet at night. No medicine.

November 23d.—No pain; the ulcer looks smaller, the pus scanty and thin. Less swelling, but some itching around the ulcer; can keep the feet in bed. No medicine.

December 5th.—Less swelling; edges less raised; healing apparent at lower edge. He has no heat in the feet and no pain in the ulcer. The itching continues. No medicine.

December 15th.—The healing at the lower edge continues; the edges look a little higher; discharge unchanged; no itching; no heat in feet. He has slight numb pain around the ulcer. He received *Silicea* (Fincke) one dose. This prescription I judge to have been a mistake; it arose from following an objective symptom, viz.: scanty discharge, against a higher, because more interior subjective indication, viz.: numbness, which would have led me to leave him under the continued action of Sulphur.

Two weeks later, on December 28th, he was no better, and felt a little short smarting in the sore, but less numbness.

I then gave him *Sulph.*$^{cm}$ (Skinner), three doses, to be taken twelve hours apart.

January 9th, 1882.—Healing advanced; scarcely at all numb; less soreness than for a long time; the pus less watery; he feels better than ever. No medicine.

January 31st.—Ulcer filled and healing much advanced; the swelling nearly gone; only a little itching; no pain; he feels better than for two years. No medicine.

February 27th.—Ulcer entirely closed; occasionally a little itching around it. No medicine.

March 23d.—Still closed; no pain; less swelling; no itching. No medicine.

April 20th.—Still healed. No symptoms. He volunteers the statement that slight wounds which formerly ulcerated now heal readily. He is discharged.

This case is one of many which illustrates the durable character of an impression by a similar drug in a high attenuation, the temporarily arrested improvement going on continuously to perfect cure for two months after the last dose of medicine.

It is an illustration of the restrictions which Homœopathy is happily placing around the art of surgery, demonstrating that many local appearances are expressions of general disorder; hence, indicating that they are by no means to be violently repelled, but cherished as signs that the organism is doing its utmost to save its most vital parts. It is an illustration of the needlessness, to say the least, of topical treatment in an inveterate skin disease, of the truth of the law of similars, and of the activity and potency of the infinitesimal.

